# Editorial: Molecular Catalysts for CO_**2**_ Fixation/Reduction

**DOI:** 10.3389/fchem.2020.00059

**Published:** 2020-02-25

**Authors:** Hitoshi Ishida, Charles Machan, Marc Robert, Nobuharu Iwasawa

**Affiliations:** ^1^Department of Chemistry, Graduate School of Science, Kitasato University, Sagamihara, Japan; ^2^Department of Chemistry, University of Virginia, Charlottesville, VA, United States; ^3^Laboratoire d'Electrochimie Moléculaire (LEM), Université de Paris, Paris, France; ^4^School of Science and Engineering, Tokyo Institute of Technology, Tokyo, Japan

**Keywords:** CO_2_ reduction, CO_2_ fixation, electrocatalysis, photocatalysis, artificial photosynthesis

Conversion of CO_2_ into useful chemicals and fuels becomes more and more important as increasing CO_2_ emissions continue to worsen the effects of climate change. Catalytic CO_2_ reduction and fixation are important components of artificial photosynthesis (the use of renewable energy to generate useful chemicals and fuels), which is vigorously studied as part of the solution to the environmental and energy problems that arise from its continuously elevating atmospheric concentration. However, the development of chemical processes remains a significant challenge for chemists. Molecular inorganic complexes, which can be utilized as homogeneous catalysts, have several advantages: well-defined structures that are readily characterized, catalytic mechanisms that can be elucidated with high fidelity, and the opportunity to optimize catalytic activities through molecular design. In this Research Topic, we present ten original research articles and three review articles dealing with electrochemical and photochemical catalysis for CO_2_ reduction or fixation.

The metal complexes, *fac*-[Mn(bpy)(CO)_3_Br] and *fac*-[Re(bpy)(CO)_3_Cl] (bpy = 2,2′-bipyridine), are known to be excellent catalysts for electrochemical CO_2_ reduction. Rotundo et al. synthesized 14 new Mn(I) and Re(I) complexes with structurally diverse 2,2′-bipyridyl derivatives and examined their catalytic activities by using cyclic voltammetry and controlled potential electrolysis. They reported that the complexes with only electron-withdrawing groups (e.g., -CF_3_, -CN) lost their catalytic activities. On the other hand, the complexes with the symmetric -NMe_2_ substituted and push–pull systems (containing both -NMe_2_ and -CF_3_) displayed electrocatalytic current enhancement. McKinnon et al. synthesized the manganese(I) complex *fac*-[Mn(bqn)(CO)_3_(CH_3_CN)]^+^ with a 2,2′-biquinoline (bqn) ligand, which had unique steric and electronic properties. They examined the catalytic activities for electrochemical CO_2_ reduction and further compared them to the efficiencies of the corresponding bpy, 1,10-phenanthroline (phen), and 2,9-dimethyl-1,10-phenanthroline (dmphen) derivatives. In the reaction mechanisms, the one-electron reduced species of the bpy complex dimerized, but the bqn ligand hindered the dimer formation to favorably afford two-electron reduced species. Talukdar et al. synthesized a new NNP-type pincer ligand, 8-(diphenylphosphaneyl)-2-(pyridin-2′-yl)quinolone, and the corresponding Fe, Co, Ni, and Zn complexes. Due to earth abundance, the development of molecular catalysts with first-row transition metals is desirable. They reported that the cobalt(II) complex showed the catalytic efficiency to produce CO in CO_2_-saturated DMF containing 5% water; the catalytic efficiencies for CO of the active complexes in descending order were Co^2+^ >> Ni^2+^ > Fe^2+^. The Zn(II) complex did not show catalytic activity. Nichols and Machan reviewed the secondary-sphere effects in the enzymes and the molecular catalysts for electrochemical CO_2_ reduction. In the CO_2_-related enzymes (e.g., carbon monoxide dehydrogenase, formic acid dehydrogenase), some amino acid residues around the catalytic centers can act as the pendent proton donors/shuttles, charged groups, etc. They discussed standard methodologies for estimating the effective catalytic overpotential and the maximal turnover frequency, and reviewed the development of secondary-sphere strategies for the classic molecular electrocatalysts, [Fe(tetraphenylporphyrin)]^+^, [Ni(cyclam)]^2+^, Mn(bpy)(CO)_3_X, and Re(bpy)(CO)_3_X (X = solvent or halide). Igarashi et al. reported the surface modification of copper cathodes with ethynyl and azide monomers. Metal copper cathodes are known to be effective for electrochemical CO_2_ reduction. The introduction of organic structures onto catalytically active metal surfaces has recently received attention because it often enhances activity and selectivity for specific products. They reported that an increase in ethylene production was observed for the organic molecule-modified copper electrodes in electrochemical CO_2_ reduction.

Photocatalytic CO_2_ reduction can be accomplished with the same molecular catalysts employed in electrochemistry by using appropriate redox photosensitizers and sacrificial electron donors instead of an electrode. The most widely used redox photosensitizer is [Ru(bpy)_3_]^2+^, which exhibits strong absorption in the visible light region and has a long lifetime in the triplet metal-to-ligand charge transfer (^3^MLCT) excited state. Tamaki et al. synthesized a new ruthenium(II) picolinate complex, [Ru(dmb)_2_(pic)]^+^ (dmb = 4,4′-dimethyl-2,2′-bipyridine; Hpic = picolinic acid), with a wider wavelength range of visible-light absorption (λ_abs_ < 670 nm) and a strong reduction ability in the one-electron reduced state (*E*_red_ = −1.86 V vs. Ag/AgNO_3_). They demonstrated that photocatalysis using [Ru(dmb)_2_(pic)]^+^ as the redox photosensitizer combined with a Re(I) catalyst reduced CO_2_ to CO under red-light irradiation (λ_ex_ > 600 nm). In contrast, [Ru(dmb)_3_]^2+^, which could not absorb light at λ > 560 nm, was not capable of working as the redox photosensitizer under the same conditions. The molar extinction coefficient is also an important factor for a redox photosensitizer. A cyclometalated iridium(III) complex having 2-(pyren-1-yl)-4-methylquinoline ligands [Ir(pyr)] has a strong absorption band in the visible region (ε at 444 nm = 67,000 M^−1^ cm^−1^) but does not act as a photosensitizer for photochemical CO_2_ reduction reactions in the presence of triethylamine as an electron donor. Kuramochi and Ishitani report that the photochemical CO_2_ reduction catalyzed by *trans*(Cl)-Ru(dmb)(CO)_2_Cl_2_ could proceed in a combination of [Ir(pyr)] as the photosensitizer and 1,3-dimethyl-2-(*o*-hydroxyphenyl)-2,3-dihydro-1*H*-benzo[d]imidazole (BI(OH)H) as the electron donor. They further synthesized a binuclear complex in which Ir(pyr) and Ru combined via an ethylene bridge. The development of efficient redox photosensitizers based on the earth-abundant metal ions as an alternative toward noble- and/or rare-metal-based photosensitizers is of continuing interest for the field. The heteroleptic Cu(I) phenanthroline complexes, such as [Cu^I^(dmp)(P)_2_]^+^ (dmp = 2,9-dimethyl-1,10-phenanthroline, P = phosphine ligand), are known to have long lifetimes and show strong MLCT excited state emission even in a solution at room temperature due to the Cu(I) center's d^10^ configuration. Yamazaki et al. synthesized heteroleptic diimine-diphosphine Cu(I) complexes, [Cu^I^(dmpp)(diphos)]^+^ [dmpp = 2,9-dimethyl-4,7-diphenyl-1,10-phenanthroline; diphos = methylene chains (*n* = 2–4) linked bis(diphenyl phosphine)]. They investigated the effects of the number of methylene chains on the photocatalytic CO_2_ reduction by using *fac*-[Re(bpy)(CO)_3_Br] as a catalyst and 1,3-dimethyl-2-phenyl-2,3-dihydro-1*H*-benzo[d]imidazole (BIH) as an electron donor. The most effective redox photosensitizer was the 1,4-bis(diphenylphosphino)butane (dppb; *n* = 4) complex. They discussed the photocatalytic results in the context of the P–Cu–P angles in the photosensitizer. Takeda et al. developed visible-light responsive Cu(I)-complex photosensitizers by introducing various aromatic substituents at the 4,7-positions of a 2,9-dimethyl-1,10-phenanthroline (dmp) ligand in a heteroleptic [Cu^I^(dmp)(DPEphos)]^+^-type complexes (DPEphos = [2-(diphenylphosphino)phenyl]ether). Introduction of biphenyl groups on the dmp ligand enhanced the molar extinction coefficient of the MLCT band. They further performed the photocatalyzed CO_2_ reduction by an earth-abundant metal complex, [Fe(dmp)_2_(NCS)_2_]. Ohtsu et al. synthesized a ruthenium(II) NAD^+^-type complex, [Ru(bpy)_2_(Me-pn)]^2+^ (Me-pn = 2-Methyl-6-(pyridin-2-yl)-1,5-naphthyridine). The common photosensitizers act as a one-electron reservoir. But they demonstrated the formation of the corresponding two-electron reduced ruthenium(II) complex, [Ru(bpy)_2_(Me-pnHH)]^2+^ (Me-pnHH (2-methyl-6-(pyridin-2-yl)-1,4-dihydro-1,5-naphthyridine) is the NADH-type ligand), by the photo-induced hydrogenation reaction of [Ru(bpy)_2_(Me-pn)]^2+^. The complex is a two-electron reservoir and will be applicable to photocatalytic CO_2_ reduction as an efficient redox photosensitizer.

Fixation of CO_2_ into organic molecules is important to obtain useful and valuable chemicals. Fujihara and Tsuji reviewed the transition metal-catalyzed carboxylation reactions of organic substrates with CO_2_ via allyl metal intermediates. They summarized the carboxylation reactions via transmetalation, catalytic carboxylation reactions using allyl electrophiles and suitable reducing agents, and then the catalytic carboxylation reactions via additional reactions affording allyl metal intermediates. Murata et al. reported the visible-light-driven hydrocarboxylation of alkenes with CO_2_ by a Rh(I) catalyst with [Ru(bpy)_3_]^2+^ (a redox photosensitizer) and BIH or BI(OH)H (an electron donor). The mechanistic study revealed that the catalysis required two photon-driven steps: the carboxylation of the hydrometalated Rh(I) complex on one hand, and the photo-induced generation of the Rh(III) dihydride complex on the other hand. The latter is an electron transfer process that requires the electron donor. The former is an energy transfer step, which would be sensitized by [Ru(bpy)_3_]^2+^. Interestingly, they found that the incorporation of the cyclometalated Ir(III) complex as a second photosensitizer with [Ru(bpy)_3_]^2+^ photosensitizer also resulted in the promotion of the reduction process. Fu et al. reviewed carbon capture and utilization (CCU) strategies. Although carbon capture and storage (CCS) strategies have been regarded as one of the promising options for controlling continuously elevating atmospheric CO_2_ concentrations, desorption and compression of CO_2_ need extra energy input. In the CCU strategy, the CO_2_ absorbents trap CO_2_ to yield the CO_2_ adducts, which can be reacted as transcarboxylation and transformation agents. For example, CO_2_ capture and Rh- or Ru-catalyzed CO_2_ adducts hydrogenation was introduced.

Conversion of CO_2_ into useful chemicals by CO_2_ reduction/fixation continues to attract significant interest and research effort. Through the remarkable creativity demonstrated by the authors, this collection of papers (and beyond) is clear evidence that CO_2_ fixation and molecular catalysis of CO_2_ reduction are and will remain very active and promising fields, promoting fundamental advances. To discover more efficient catalysts, mechanistic investigations on ligand effects, as well as the modification of the second sphere in the metal complexes, will continue to expand the impact of these efforts, with the help of many various complementary *in situ* spectroscopic techniques, such as IR, Raman, EPR, mass, and X-ray absorption spectroscopies. Developing new hybrid systems, such as those that combine the most promising molecular catalysts with semiconductor materials, will also be important developments in the near future, merging the best attributes of homogeneous and heterogeneous catalysis. We hope that this Research Topic will contribute to stimulating the next wave of research efforts in the construction of new artificial photosynthetic systems.

## Author Contributions

All authors listed have made a substantial, direct and intellectual contribution to the work, and approved it for publication.

### Conflict of Interest

The authors declare that the research was conducted in the absence of any commercial or financial relationships that could be construed as a potential conflict of interest.

